# Human Grasp Mechanism Understanding, Human-Inspired Grasp Control and Robotic Grasping Planning for Agricultural Robots

**DOI:** 10.3390/s22145240

**Published:** 2022-07-13

**Authors:** Wei Zheng, Ning Guo, Baohua Zhang, Jun Zhou, Guangzhao Tian, Yingjun Xiong

**Affiliations:** 1 College of Artificial Intelligence, Nanjing Agricultural University, Nanjing 210031, China; zhengwei583@stu.njau.edu.cn (W.Z.); 2021282120116@whu.edu.cn (N.G.); xyj@njau.edu.cn (Y.X.); 2 College of Electronic Information, Wuhan University, Wuhan 430061, China; 3 College of Engineering, Nanjing Agricultural University, Nanjing 210031, China; zhoujun@njau.edu.cn (J.Z.); tgz@njau.edu.cn (G.T.)

**Keywords:** agricultural manipulator, tactile glove, manual simulation, grasp analysis, grasp planning

## Abstract

As the end execution tool of agricultural robots, the manipulator directly determines whether the grasping task can be successfully completed. The human hand can adapt to various objects and achieve stable grasping, which is the highest goal for manipulator design and development. Thus, this study combines a multi-sensor fusion tactile glove to simulate manual grasping, explores the mechanism and characteristics of the human hand, and formulates rational grasping plans. According to the shape and size of fruits and vegetables, the grasping gesture library is summarized to facilitate the matching of optimal grasping gestures. By analyzing inter-finger curvature correlations and inter-joint pressure correlations, we investigated the synergistic motion characteristics of the human hand. In addition, the force data were processed by the wavelet transform algorithms and then the thresholds for sliding detection were set to ensure robust grasping. The acceleration law under the interaction with the external environment during grasping was also discussed, including stable movement, accidental collision, and placement of the target position. Finally, according to the analysis and summary of the manual gripping mechanism, the corresponding pre-gripping planning was designed to provide theoretical guidance and ideas for the gripping of robots.

## 1. Introduction

Agriculture is fundamental to human survival, and skilled agricultural labor is gradually becoming one of the most demanding factors in the industry [1]. However, the increasing trend of population aging has caused a labor force shortage for agricultural work. Compared with human beings, robots have superior durability and high repeatability, and can replace or supplement humans to complete tedious and dangerous tasks, which provides a potential solution to the above problems [2,3]. With the high requirements of automation brought by the industrial upgrading of the agriculture and food industry, the application of agricultural robots will be more extensive. As a result, there has been a growing interest in using agricultural robots to pick, sort, and pack fruits and vegetables over the past three decades [4,5]. The development of such platforms involves many challenging tasks. Grasping, moving, and placing objects are the essential functions and common operations of robots and manipulators [6]. Since fruits and vegetables are often fragile, irregular, and slippery, a non-destructive, stable, and firm grasping target remains a challenge for the manipulator [7,8,9]. Previous studies have amply proven that appropriate grasp configuration and reasonable gripping planning are the key factors in ensuring the safe and steady grasping on target objects [10,11].

In many agricultural tasks, potential slippage and damage risks should be avoided when grabbing and placing objects due to the large differences in shape, hardness, and surface properties of agricultural products [12,13]. Thus, the proper grasping configuration (that is, the gripping posture of the hand relative to the object) is necessary for the grasping of the manipulator. Humans can use their hands flexibly to complete various complex tasks. Understanding the characteristics and mechanism of human grasping has important guiding significance and reference value for humanoid robotic grabbing. Understanding how humans grasp fruits and vegetables, understanding the kinematic implications and limitations associated with each grasp, and understanding common usage patterns are important in many areas of agricultural picking, sorting, and packaging. In gripping tasks, it is important to understand not only the gripping shape for normal use, but also how to adapt the gripping posture to the task requirements. Each grip can be classified according to the need for precision or power, and the hand posture is very important and influences many authors. Numerous studies on hand grasping bypass the more complex biological structure, and the approach of hand grasping posture as the entry point for research is widely adopted. According to the shape characteristics of hand grasping and grabbed objects, Schlesinger [14] divided hand grasping posture into six categories from the functional aspect, including fingertip pinch, side pinch, clamp pinch, hook, spherical grab, and cylindrical grab. Then, Napier [15] pointed out that the surface characteristics, size, shape, and other factors of the target object will directly affect the grasping motion and divided the grasping configuration into the precision grip and power grip. Iberall [16] first proposed the concept of the virtual finger (VF), in which one or more fingers were classified as the same functional unit by applying force in similar directions and acting in a consistent manner in the process of grasping [17]. Grasping posture can thus be divided into three basic types: lateral finger confrontation, interfinger confrontation, and palm confrontation. According to different classification basis, Vergara et al. [18] and Feix et al. [19] divided the grasping posture into 9 and 33 categories, respectively. In addition to crawling configuration, reasonable and effective grasping planning is also necessary to ensure robust and successful grasping.

Compared with robots, humans are adept in holding target objects flexibly and robustly in different backgrounds when performing grasping tasks. Although great progress has been made in the research of manipulator gripping, achieving the same level of robustness and versatility as the human hand is still difficult [20]. Therefore, designing and controlling the manipulator based on the analysis of manual grasping characteristics is essential for the intelligent planning of the manipulator [21]. Starke et al. [22] investigated the force synergy effect in human grasping behavior and further understood the hand dynamic properties of grasping motion by analyzing the correlation between the grasp forces in fingers and palm. In addition to force synergy, Naceri et al. [23] focused their research on gripping force control. They investigated unconstrained hand gripping in the framework of motor synergy theory to reduce motor redundancy and achieve stable gripping. When the manipulator performs grasping task, it needs to interact with the external environment constantly through various sensors [24]. Additionally, the establishment of a multi-sensor fusion feedback network plays a crucial role in ensuring the stability of the target work. With the deepening exploration of the human-hand grab mechanism, the analysis of grasping characteristics based on tactile information is particularly important. Coupled with the continuous progress of sensors and sensing technology, tactile sensors are widely used in the research of grabbing analysis. Nicholas et al. [25] applied pressure sensors in their research to investigate the relationship between the force and contact area of a cylinder operated by human hands. Li et al. [26] designed a skin-inspired quadruple tactile sensors which can integrate the information of pressure, object thermal conductivity, object temperature, and environment temperature. Its application to robot grippers enables accurate recognition of objects of different shapes, sizes and materials. Battaglia et al. [27] developed a wearable fingertip force/moment sensor, which can be used in grasping analysis to accurately measure the contact force between the hand and the target object to further investigate the sensorimotor control. Sundaram et al. [28] combined tactile gloves with the sensor array and deep learning technology to recognize target objects and explore tactile patterns. The extended sensor array can capture tactile information from almost the entire hand. Many related studies have also realized data acquisition, object recognition, and grasping pattern identification based on tactile perception [29,30,31].

Although previous research on tactile perception and grasp configuration have been carried out, the studies mentioned above are generally only aimed at specific research directions. There are few studies on grasp configuration and grasp planning based on the analysis of manual grasping patterns and haptic perception. Thus, the main purpose of this paper is to study the mechanism during manual grasping, so as to provide theoretical guidance for the development of grasping plans for agricultural manipulators that need to be involved in fruit and vegetable grasping, such as fruit and vegetable picking and sorting. Specific objectives include the following: (1) design and develop the tactile data collection glove, and complete related grasping experiments; (2) summarize the grasping posture library according to manual grasping characteristics and experience; (3) combine the haptic information to analyze the inter-finger curvature correlation and inter-joint pressure correlation; (4) investigate the slippage occurrence and external acceleration interaction during gripping; (5) analyze the multi-finger grasp strategy and make rational grasping planning to provide theoretical guidance for the manipulator.

## 2. Materials and Methods

### 2.1. Physiological Structure of the Human Hand

**Figure 1:**
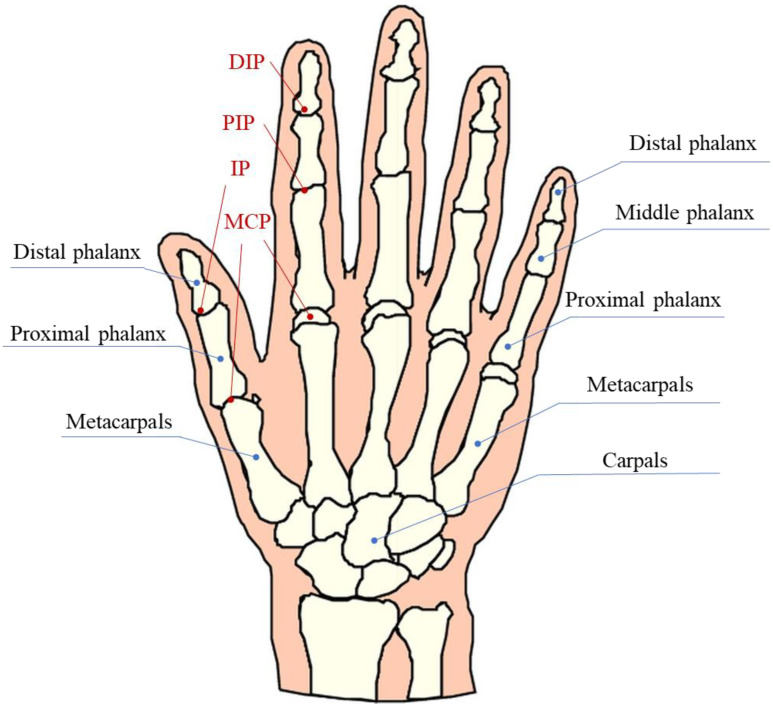
Physiological structure of the human hand [33].

The human hand is the optimum reference object for manipulator design and development [32]. Whether from the structure, function, operational coordination, and other aspects, the performance of human hand is the supreme target for bionic manipulator research. Analyzing the physiological structure of the human hand is the basis for exploring its grasping mechanism and providing ideas and guidance for the design, movement, and control of the manipulator. Generally, a human hand consists of five fingers, including the thumb, index finger, middle finger, ring finger, and pinky. Except for the thumb, which has only two knuckles, the other fingers are all three knuckles. As shown in Figure 1, according to the proximity from the palm, the phalanges are named as the distal phalanx, the middle phalanx, and the proximal phalanx, respectively. Similarly, the joints between them are distal inter-phalangeal (DIP), proximal interphalangeal (PIP), and metacarpal phalangeal (MCP), respectively. Moreover, the structure connecting the proximal phalanx to the wrist is the metacarpal bone, which constitutes the main part of the palm. Human fingers mainly include the following motions: bending and extension, outreach and inward movement, and ring rotation movement. Although the thumb lacks a knuckle structurally compared with other fingers, the movement is much more complicated. Not only can the thumb perform flexion and extension movement, but also its inward/outreach, as well as the opening and closing movement to the palm is incomparable to other fingers. In addition to flexion and extension, the three joints of the remaining four fingers can only perform small inward/outreach movements.

### 2.2. Grab Force Closure and Stability

When agricultural robots perform picking or sorting tasks, it is always expected that the end manipulator can grasp steadily. Once the robot arm has moved to the target position, a safe and stable force analysis is a precondition to avoid damage to the object caused by excessive grasping force or the object slipping due to too little gripping force. Due to the structure, control conditions, and sensitive perceptual restrictions, the manipulator cannot complete that flexible work as a human hand. Therefore, to make up for the inherent deficiencies of manipulator, attention must be paid to the analysis of grasping stability and thus formulate an effective grasping plan.

**Figure 2:**
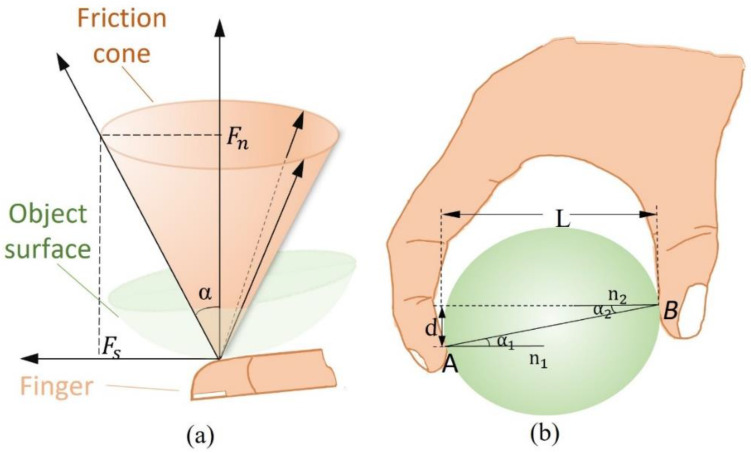
Friction cone and the two-finger grasping model. (**a**) Schematic representation of the friction cone in grasping. (**b**) The two-finger grasping model.

The grasping stability is usually studied by force and form closure theories depending on whether friction cone constraints are involved in the grasping task. Friction cone is a fully binding rotating vertebra synthesized by normal binding $F_{n}$ and static friction force $F_{s}$ in an instant of relative sliding of the object (the axis is the common normal, as shown in Figure 2a) [34]. Form closure is a purely geometric property without considering the effect of grip force on task stability. This section analyzes the conditions for stable grasping based on the force closure principle. Force closure is a state in which certain conditions must be met for the contact between the finger and object for the object to reach static equilibrium or relative palm movement of freedom is zero [35].

When multiple fingers participate in grasping, according to the Coulomb friction theorem and the force vector in Figure 2a, it can be known that the requirements for avoiding sliding and maintaining the stability of contact points need to meet the following formula:

(1) $$F_{s}<\mu F_{n}\;$$

The above formula indicates that the static friction force at the contact point must be on the surface or inside of the friction cone, and the angle (friction angle) between the full binding force and the normal binding force is maximum when it is located on the surface. Additionally, the friction angle satisfies:

(2) $$tan\alpha =\frac{F_{s}}{F_{n}}=\mu$$

For different contact forms, the representation of the friction cone changes depending on the force constraints.

For the friction-point contact:

(3) $$FC=\left\{f\in R^{3}:\sqrt{f_{x}^{2}+f_{y}^{2}}\;\leq \mu f_{z},f_{z}\geq 0\right\}$$

For the soft-finger contact:

(4) $$FC=\left\{f\in R^{4}:\sqrt{f_{x}^{2}+f_{y}^{2}}\;\leq \mu f_{z},f_{z}\geq 0,\left|m\right|\leq \gamma f_{z}\right\}$$

where f is grasping force, $f_{z}$ is normal binding force, $f_{x}$, $f_{y}$ is the tangential force of f. μ, m, γ are coulomb friction coefficient, normal torque, and torque friction coefficient, respectively [36,37].

Thus, the multi-finger contacts conditions based on force closure include normal binding force unidirectional, the contact point inside the friction cone and satisfying the force balance condition.

When only two fingers participate in grasping, the contact model is shown in Figure 2b. A, B are the two contact points, *n*_1_ is the normal vector at point A, *α*_1_ is the angle between the line AB and the normal, and the same is true at point B [38].

According to the contact model, the following formula is obtained:

(5) $$\alpha_{1}=\alpha_{2}=\mathrm{arctg}\left(\frac{d}{L}\right)$$

where *d* is the vertical distance between two points of A and B, and *L* is the distance of the horizontal projection of the line AB. Therefore, the contact between two fingers meets the conditions of force closure:

(6) $$\alpha >\alpha_{1}=\alpha_{2}$$

In conclusion, the stability analysis of contact forces is a prerequisite to ensure robust grasping. Exploring the multi-finger and two-finger gripping laws based on the force closure principle contributes to rational gripping planning and gripping control of manipulators.

### 2.3. Grasping Posture Analysis

**Figure 3:**
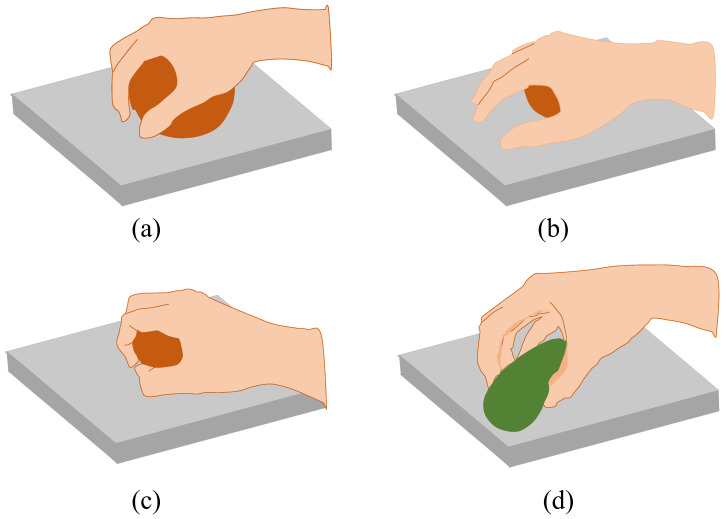
Typical hand grasping posture. (**a**) The palm-aligned power grasping. (**b**) The palm-aligned grasping for small diameter objects. (**c**) The proximal phalanx-aligned grasping. (**d**) The precision grasping.

Currently, the in-depth studies on the analysis of manual grabbing modes have been extensive. This study summarizes the grasping patterns of fruits and vegetables with different shapes and sizes in agricultural scenarios. Since most fruits and vegetables are convex and relatively regular in shape, they can be classified into three categories according to their geometric characteristics: spheres, cones, and cylinders, which can be referred to analyze the appropriate grasping mode. The spherical, cylindrical, and conical types of fruits and vegetables are the most common and representative and are the ones most studies choose to study. These shapes make up the vast majority of fruits and vegetables. Spherical products have the characteristics of the symmetrical structure and smooth surface curvature. As shown in Figure 3a, the five fingers combined with the palm for power grasping can stably envelop the target object in the largest range. However, due to the constraints of the geometric radius of the finger and the object, not all spheres are suitable for this method (as shown in Figure 3b). When the target object is small in diameter, using the power grip of palm and fingers can cause the fingers to collide with the table during bending and thus unable to bend to fit the surface of the object. Therefore, the power grip is subdivided into a palm-aligned power grip and a proximal phalanx-aligned power grip for spherical target objects, as shown in Figure 3.

The gripping mode is determined by analyzing the relationship between the radius of the target object and the length of the finger. The corresponding grasping posture is shown in Table 1. Suppose the middle finger length is L and the object radius is r, when L<πr/3, the palm-aligned power grasp method will be used to envelop the object. When πr/3<L<2πr/3, the proximal phalanx-aligned power grasp is adopted, which uses the proximal phalanges to replace the palm function and increases the contact length between the fingers and the object. For the objects with L>2πr/3 such as kumquats or saints, a precision gripping pattern such as the two-finger pinch can be used, and the clamping angle is bounded by the diameter of the target object. Cylindrical fruits and vegetables have a circular cross-section. For larger volumes, such as cylinders with a cross-sectional radius satisfying L<πr/3 and the product length satisfying s>width (width of mechanical palm), the palm-aligned five-finger power gripping can be performed. Additionally, when s<width, the *n* + 1 finger-palm-aligned power grasping is performed according to its length ratio (n=5s/width, where width/5 is approximately equal to the width of the middle finger). Similarly, when r satisfies πr/3<L<2πr/3 and is captured in the same way as above. For cylindrical products, the thumb is often positioned at the center of the cylinder to reduce the risk of slipping caused by the unstable and tilting center of gravity of the object during the grabbing process. For smaller-sized cones of fruit and vegetables, such as strawberries, the three-finger pinch pattern can be used.

### 2.4. Tactile Glove and Multi-Sensor System

#### 2.4.1. Tactile Glove Design

**Figure 4:**
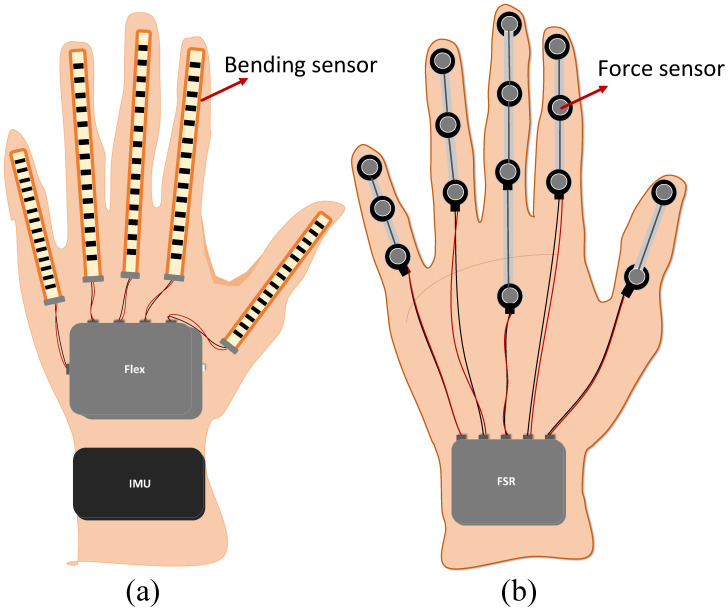
The tactile glove and sensor distribution. (**a**) Distribution of bending sensors and IMU on the back of hand. (**b**) Distribution of pressure sensors in palm.

The human hand can perceive external objects and perform various operations. Meanwhile, hand grasping can be regarded as a composite operating system integrating sensors and clamping tools [5]. The tactile glove designed in this paper was used to collect various haptic information in manual grasping simulation analysis. The data acquisition glove takes the canvas glove as a carrier and fixes the sensor group on it. The specific sensors distribution can be referred to in Figure 4, five bending sensors are attached to the back of fingers, which effectively constitutes the bending sensing module. Similarly, in order to measure the grasping force, eleven pressure sensors are installed at the corresponding part of the finger belly and form the pressure sensing module. The reasonable layout of the sensors makes the user wearing tactile glove to grab the target object without affecting the normal movement of the hand joints. As a wearable virtual reality medium of exchange, the tactile glove is responsible for touch perception and sending collected data to the computer.

#### 2.4.2. Bending Sensing System

The Flex 2.2 flexible bending sensors with the length of 2.2 inches were adopted in the experiment. To prevent interference with the perception of the force sensor, they are equipped on the upper side of the five fingers of the data glove. The resistance value of the bending sensor changes depending on the bending. When the substrate is in the bending state, the resistance output depends on the bending radius, and the smaller the radius, the higher the resistance value. Additionally, when it is in a horizontal non-bending state, its resistance value is about 9k ohms. When the sensor is bent at 90 degrees, the resistance is about 14k ohms. When it reaches 180 degrees of bending, the corresponding resistance is about 22k ohms. The bending sensing circuit on the glove is connected to the BLE4.2 Bluetooth circuit, and the computer reads the real-time curvature via Arduino.

#### 2.4.3. Force Sensing System

Proper grasping force is a key factor for safe fruit grasping without potential slippage and damage. The fifteen force sensors applied in this study are FSR (Force Sensing Resistors) resistive film pressure sensors with the model of IMS-C10A. As shown in Figure 4, they are arranged at the abdomen and palm of the tactile glove, which helps to make the forces-sensing module cover almost the entire palm. The FSR sensor comprises two molecular films (PVDF film) separated by a thin air gap and has the advantage of small size and ultra-thin without affecting the hand movement when grabbing. As a series of FSR, IMS (I-Motion) is a single-area force-sensitive resistor whose output resistance decreases as the pressure on the effective surface increases. The FSR series are capable of measuring both dynamic and static forces exerted on contact surface as well as providing favorable active compliant control. More information about FSR sensors can be found in a compendium of Sadun et al. [39].

#### 2.4.4. IMU Interactive Perception System

Inertial Measurement Unit (IMU) is a medium for measuring the axial attitude angle (or angular rate) and the acceleration, which can obtain information about the velocity, posture, and displacement of the carrier. The IMU placed at the wrist is a HWT906 nine-axis attitude sensor containing three monoaxial accelerometers, gyroscopes as well as magnetometers to measure three-dimensional acceleration, angular velocity, magnetic field, and angle. For the gripping and placing operation of the robot, it is necessary to perceive the interaction between objects and the external environment, such as the object has been placed in a specified position or colliding with an obstacle in the grasping process. When performing the grasp operation, IMU mostly combines with other sensors to realize the position and state judgment of the hand, further improving the grasp control and motion planning of the manipulator. In this study, the IMU was applied to collect the variation information of the acceleration signal during an experiment, which is helpful to understand and judge the interaction as well as provides a theoretical basis for the grasping judgment of the robot.

## 3. Results and Discussion

### 3.1. Finger Correlation and Cooperation Analysis

The human hand has always been the best source of inspiration for manipulator development, and the kinematics as well as dynamics of hands are the research focus of grasping behavior. In the grab operation, the hand fingers are coordinated with movement and cooperate differently for different target objects. In many tasks, multiple fingers will work collaboratively as the same functional unit (virtual finger (VF) for manipulator). Investigating the work allocation between fingers can not only benefit the selection of optimal grasping mode, but also facilitate the optimization of anthropomorphic movement and cooperative control of the manipulator.

#### 3.1.1. Curvature Correlation

**Figure 5:**
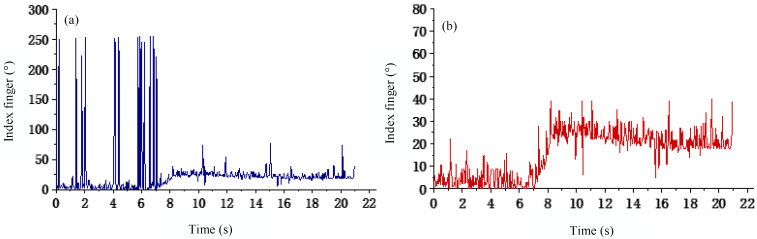
Index finger curvature. (**a**) Original data of curvature. (**b**) Preprocessed bending data.

Therefore, in this paper, the correlation and partnerships of the human fingers are explored by the grasping experiments, including the bending correlation between fingers and the pressure correlation between joints. The experimental procedure is as follows: Wear data gloves to grasp each group of fruit placed on the plane. Specific actions include spreading the palm near the target object, bending the fingers for envelope grasping (or pinching), lifting the target object for five seconds and keeping it stable, placing the object in another position in the plane, and ending the grasp. For different diameters of fruits and vegetables, the bending degree of the five fingers under the stable grasping condition is shown in Table 2. To observe the finger coordination manner more intuitively, the representative grasping with all five fingers involved is chosen in the following analysis. Figure 5 shows the bending information of the five fingers for an apple (sphere) with the diameter of 8.4 cm captured by the enveloping method. As can be seen from Figure 5a, a lot of noise exists in the original curvature data. Therefore, it is necessary to de-noise the experimental data before analyzing the correlation of finger curvature, the denoised picture is presented in Figure 5b.

After reading in the data from the bending sensors and obtaining the bending value in the steady grasp state after signal processing, the angle of each finger was formed into a set of data. Suppose there are two data sets, sequence A and sequence B, where $\bar{A}$ and *A_i_* are the average value and the *i*th value of sequence A, respectively (the same with B). Then, the correlation coefficient *r* of the two data sets can be calculated by the following formula:

(7) $$r=\frac{\sum_{i=1}^{n}\left(A_{i}-\bar{A}\right)\left(B_{i}-\bar{B}\right)}{\sqrt{\sum_{i=1}^{n}{\left(A_{i}-\bar{A}\right)}^{2}}\sqrt{\sum_{i=1}^{n}{\left(B_{i}-\bar{B}\right)}^{2}}}$$

**Figure 6:**
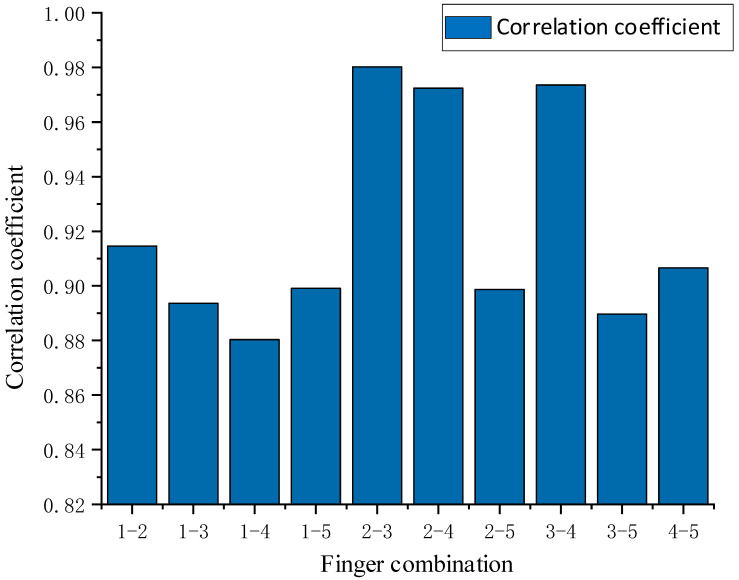
Correlation coefficient of five-finger curvature.

The histogram of curvature correlation between different fingers is shown in Figure 6, where 1 represents the thumb, 2 the index finger, 3 the middle finger, 4 the ring finger, and 5 the little finger. The calculation results illustrate that the correlation coefficient between the thumb and other four fingers is relatively lower, indicating that the thumb is more independent in its movement. This can be a difficulty in researching the coordinated motion of the manipulator because the thumb has higher freedom and is more engaging than the other fingers. Moreover, the thumb is also the most flexible of all fingers with the widest range of fingertip workspace. Similarly, the little finger showed a lower correlation. This may be related to the power provision and object support of the little finger in grasping objects, which plays an important stabilizing role to ensure the stability and balance of the holding object. Conversely, the highest correlation is found between the index, middle, and ring fingers. The phenomenon suggests that the synergistic motion between these three fingers is the most significant in human grasping operation. The effective cooperation and association between fingers during stable handgrip can also be used as one of the judgment conditions for a stable grip. Therefore, referring to the curvature correlation between the fingers, the virtual fingers can be allocated reasonably in the grasp control and planning of the manipulator. By controlling the collaborated movement between the interconnected fingers, a better manipulator grip can be controlled.

**Figure 7:**
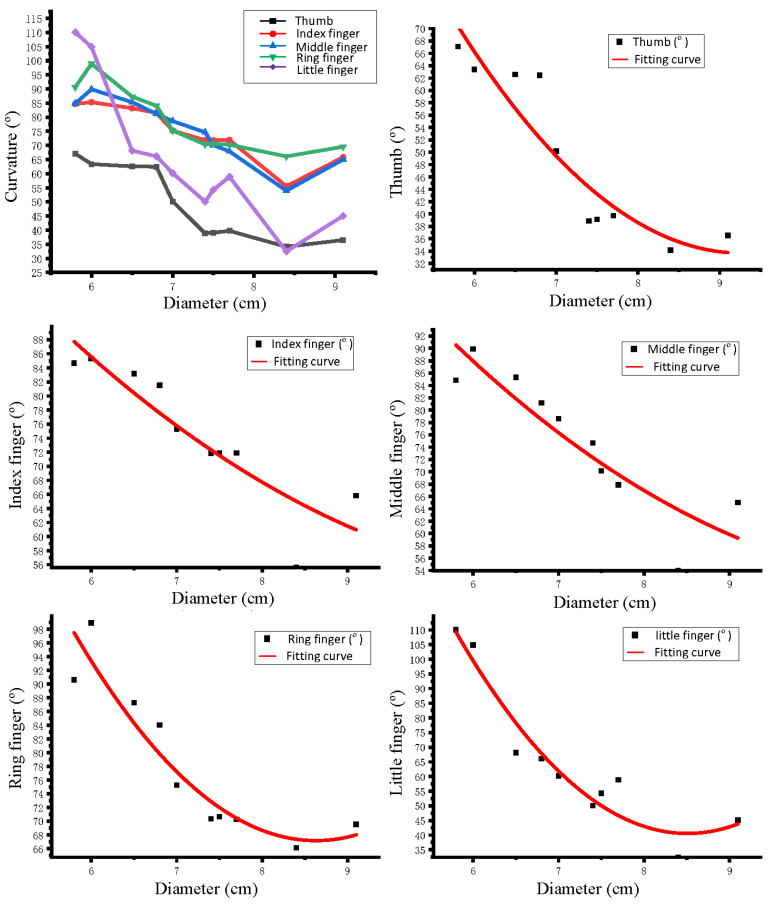
Fitting relationship between finger curvature and diameter.

To investigate the relationship between the change in curvature and the diameter of the object during manual grasping, the functional relationship between the five fingers and the diameter was derived by the second-order polynomial fitting. This function can be used as the initial value of the curvature to guide the grasping of the manipulator. According to the fitting function image (Figure 7), as the diameter of the object increases, the finger curvature decreases, which is consistent with the actual situation.

#### 3.1.2. Force Correlation

**Figure 8:**
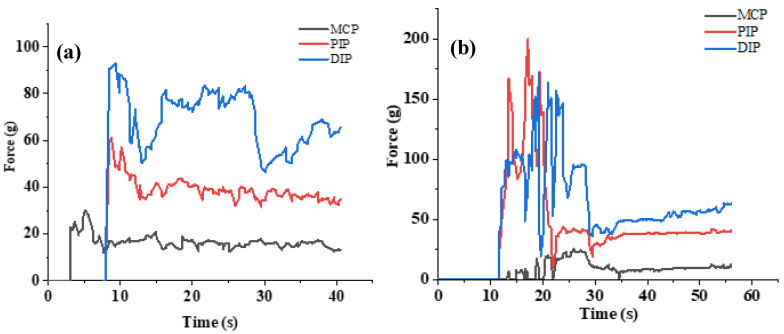
Pressure variation at the three joints of fingers. (**a**) Pressure changes on the three joints of the middle finger. (**b**) Pressure changes on the three joints of the little finger.

In addition to collecting the bending information during the grasping process by the bending sensors, we also collected the force signals of each joint in a grasping experiment by the force sensors. Figure 8 shows the force signals of the three knuckles of the middle finger and little finger in a certain hand-grabbing simulation. Through many experiments, it is found that the force at MCP is the lowest among the three joints in the stable grasping state. The relative force of PIP and DIP joints is higher, and the force of the DIP joint is basically greater than or equal to the force at PIP. The pressure relationship between the three finger joints can be used as an initial judgment condition for stable grasping. Similarly, by solving the correlation coefficient of grasping force of three joints, the knuckle force correlation in the manual grasping process can be obtained. As can be seen from Table 3, the independence of MCP joint force is the highest overall, and the index, middle, ring, and little fingers all meet the highest PIP-DIP force correlation. In fact, the index finger, middle finger, and thumb usually provide the pinching and gripping power, while the ring finger and little finger cooperate in providing the gripping power. The tacit cooperation between fingers and knuckles is also a key factor to ensure stable grasping.

### 3.2. Slippage Detection

**Figure 9:**
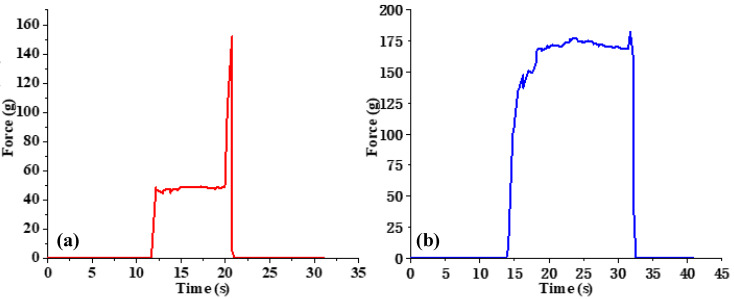
Slippage detection based on force signal. (**a**) The target object is stably placed on the target sensor. (**b**) The target object slides away from the pressure sensor.

**Figure 10:**
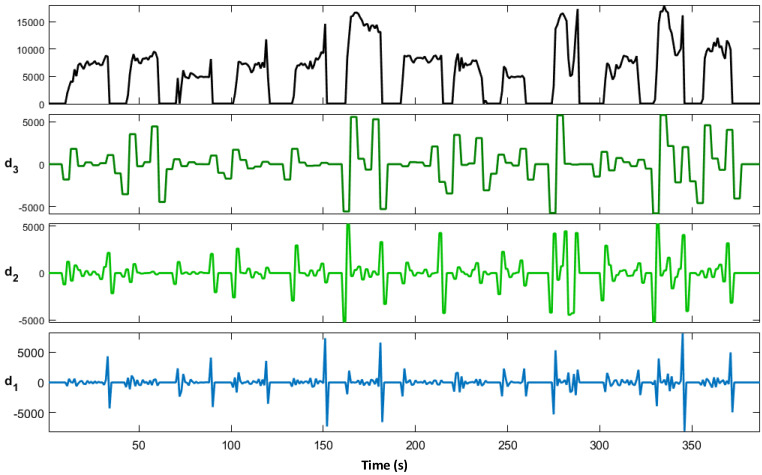
Pressure signal after wavelet transform.

Since fruits and vegetables generally have diverse shapes and are fragile, the appropriate grasping force is particularly crucial to secure and avoid potential slippage. Insufficient grasping force may easily lead to slippage, at which point the pressure will generally continue to increase, but excessive pressure often causes fruit damage. Thus, proper pressure is a necessary condition for safe grasping. Humans can obtain interactive information based on the force receptors on the skin surface and adjust the grasping force timely according to the actual situation, which is a challenge for robots. Therefore, slippage detection is an important prerequisite for robust crawling and timely adjustment of grasping force. The slippage occurrence can be detected based on the force signal. As shown in Figure 9, the output pressure changes relatively stable when the object is statically placed on the force sensor, while when the object slides, its pressure value will generate an instantaneous pulse. Eventually, when the object slips to separate from the force sensor, the pressure value rapidly decreases to zero, and the slip signal is obvious. However, due to irresistible human factors and environmental factors, the force data will fluctuate and cause interference when the pressure information is collected by wearing the data glove, which makes it difficult to analyze the sliding signals from a single time domain. Studies have shown that the sliding phenomenon is accompanied by the high-frequency electrical signals output from the pressure sensor, and the high-frequency component can be an important factor in judging whether the slip occurs. Currently, common methods for efficient acquisition of high-frequency signals include Fourier transform, high-pass filter, wavelet transform, and so on. In these methods, such as high-pass filters, s difficult to select appropriate cutoff frequency to distinguish interference frequency from slip frequency. The Fourier transform has theoretically infinitesimal resolution in the frequency domain, but it also loses all the time domain information and suffers from problems such as the inability to locate the time period when the slip occurs. Moreover, Fourier transform has a high requirement on the accuracy of the original signal, and its image in the frequency domain is easily affected by external interference. Therefore, Fourier transform is not strong in analyzing non-stationary signals. However, the wavelet transform can obtain the time-frequency domain information and has a high frequency resolution for the characterization results of the low-frequency components of the analyzed signal, and a high time resolution for the characterization results of the high-frequency components. Additionally, it achieves better performance for sudden and drastic distortion signals such as slip signals with attenuated finite length wavelet bases. Therefore, in this study, we choose wavelet transform to judge the occurrence of slip. In Figure 10, d1, d2, and d3 are the first-order, second-order, and third-order wavelet transform results of the original voltage signal, respectively. It can be found that with the generation of slippage, the wavelet transform shows clear coefficient peaks, which can judge the occurrence of sliding during gripping by setting the amplitude threshold.

### 3.3. External Interaction Judgment

For the grasping and placing operation of the robot, it is necessary to perceive the interaction between the object and the external environment, such as the object has been placed in a specified position or collided with obstacles during the grasping process. This paper analyzes the acceleration signal changes during the grasping operation and tries to understand and judge the interaction, thus providing a theoretical basis for the grasping judgment of the robot. In this section, we launched two related experiments, one is the desktop interaction experiment, and the other is the obstacle collision experiment. The specific procedure of the desktop interaction experiment is as follows: attach the IMU sensor to the wrist and keep the hand slowly close to the target object. Then, hold the object and lift it 15cm high, keep the vertical height unchanged and move evenly to the target position. Finally, drop the object at a constant speed until it contacts with the table. Similarly, the first two steps of the obstacle collision experiment are the same as the desktop interaction experiment. Then, simulate the action of encountering the preset obstacle in the Y and Z directions and stop the movement. It is worth stating that the precision and accumulated error of IMU is indeed an issue that should be considered if the fine-grained operation or movement trajectory of human hand is studied. However, in this paper, the main purpose is to study the mechanism during manual grasping, so as to provide theoretical guidance for the development of grasping plans for agricultural manipulators that need to be involved in fruit and vegetable grasping, such as fruit and vegetable picking and sorting. Therefore, there is no high requirement for the accuracy of IMU, it only needs to be able to reflect the movement pattern and response action characteristics of human hands, and the accuracy of the IMU used now is sufficient to complete our research.

**Figure 11:**
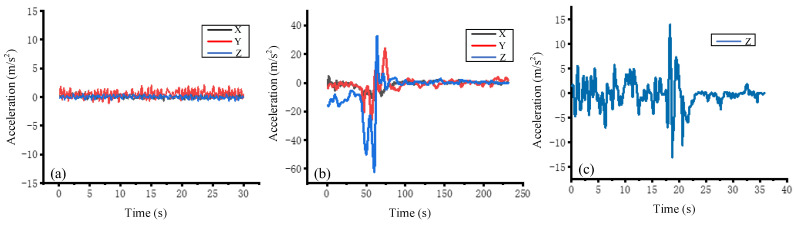
External interaction perception based on acceleration. (**a**) The hand maintains static or move at a uniform speed. (**b**) Collision with obstacles located in the Y and Z axes. (**c**) The change in Z-axis acceleration when the object is being placed on the table.

The experimental results are shown in Figure 11. When hands maintain static or move at a uniform speed, the acceleration in X, Y, and Z directions is 0 (Figure 11a). As shown in Figure 11b, the acceleration in the Y and Z directions produces large perturbations, while the acceleration transformation in the X direction changes relatively gentle. So, it can be judged that the obstacle is located in the Y and Z axis of the object, and there is no obstacle in the X-direction. When a collision occurs during grasping, the acceleration amplitude in the direction of the collision will fluctuate greatly, which can serve as a symbol of collision detection. Figure 11c shows the acceleration change of the Z-axis when the object was placed on the desktop after the grasping operation was completed. At the 18 s point, the object collides with the desktop, and the acceleration increases instantly. The analysis above facilitates replanning the grasping path after the collision of mechanical grasping.

### 3.4. Grasping Control and Planning for Manipulator

According to the analysis and relevant conclusions in the preceding sections, the control and planning of manipulator grasping are carried out based on the understanding of manual grasping mechanism. The following content makes a general plan for the manipulator grasping mainly from three parts: gripping configuration and bending control, pressure perception and slippage detection, and interaction judgment. In the grasping configuration stage, the robot will first select the grasping pattern according to the geometric shape and feature size of the target object. It includes grasping posture (power grasping or precision grasping), effective mechanical finger allocation (actual number of fingers involved in the grip), and virtual finger planning. A reasonable allocation of virtual fingers can improve the cooperative motion mechanism of each finger and reduce the complexity of motor control. Combined with the grasping gesture library collated in Section 2.2, the corresponding virtual finger assignments are shown in Table 1, where T, I, M, R, and L represent the thumb, index finger, middle finger, ring finger, and little finger, respectively. Once the pattern is determined, the robotic arm will move to the target position with the manipulator ready to grasp.

**Figure 12:**
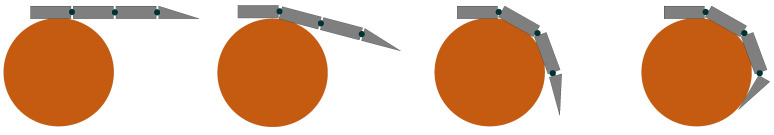
Control sequence of each joint of manipulator.

After the preparation grab, it enters the bending control stage. The relative task is to set the initial value of mechanical grasp curvature based on the geometry, radius, and the curvature model established with the previous grasp analysis. When the contact signal appears as the manipulator approaches the target object, the mechanical finger bends to the specified initial bending value according to the signal generated in different situations. The contact signals here can be divided into three categories, the first of which is the palm-type power grip contact signal. In this case, the data M of the pressure sensor at the palm is used as the trigger symbol, and when M is unequal to 0, the contact has been triggered. According to the law of hand grasping, the manipulator successively bends the MCP, PIP, and DIP, and the control sequence of each joint is shown in Figure 12. The second category is the power grasp of the proximal interphalangeal joint, with the pressure signal MP of proximal interfinger joint as the trigger signal. When the MP is nonzero, the remaining two joint can be bent for grasping. The last one is precision grasping. When the pressure signal 1_PIP at the distal knuckles of the thumb is nonzero, indicating that grasping can be performed. Then, the index finger (or effective fingers assigned according to the actual situation) will be bent to cooperate with the thumb for force confrontation to achieve an accurate pinching operation.

After the mechanical finger makes full contact with the object, the surface force will be sensed through the FSR pressure sensor. According to the research in Section 3.1, there is a certain correlation between the forces of the three joints in a stable grasping state. The pressure decreases sequentially from the distal phalangeal joint to the metacarpal joint, which can be used as an initial judgment for attempted grasping. Once the joint force meets the initial grasping criteria, the robotic arm moves upward, and the manipulator tries to grab objects. When power grasping is adopted, the trigger flag (M, MP) will jump to 0 instantaneously at the moment of grasping to trigger the wavelet transform algorithm. In the precision capture mode, the wavelet transform can be triggered when the 1_PIP joint is nonzero. The signal obtained by the pressure sensor is processed with the wavelet amplification algorithm. When the threshold exceeds the set sliding threshold, it is judged that sliding occurs. Meanwhile, the motor is continued to increase the bending degree of the mechanical finger, thereby increase the grasping force. When the threshold is less than the set sliding threshold, it is judged that the slip is terminated, and the manipulator immediately stops the execution of the action and no longer increases the grasping force. Therefore, the manipulator will not continuously increase the gripping force, so as to ensure the nondestructive grasp of fruits and vegetables.

**Figure 13:**
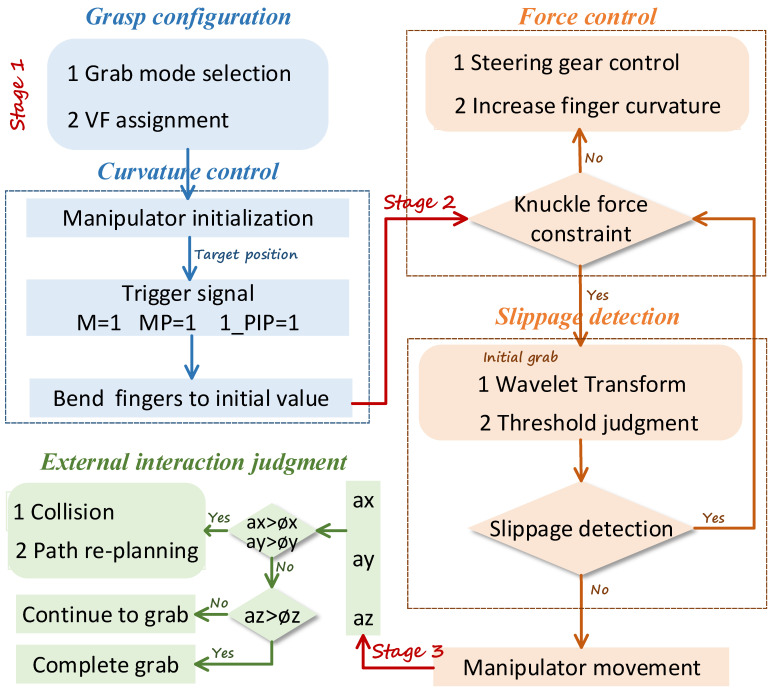
Grasp planning flow chart of manipulator based on manual grasp mechanism analysis.

Finally, it is crucial to judge the interaction between the manipulator and the outside environment by the IMU sensor. The acceleration of XYZ axes can reflect the motion state of the manipulator. When the z-axis directional acceleration exceeds the collision threshold, it indicates that the grasping operation has been completed and the object is placed on the desktop. An obstacle collision was estimated to have occurred when the acceleration in ax, ay is greater than the collision threshold, and then the path is re-planned in reverse motion in accordance with the original motion direction of the manipulator. All the above control selection and planning processes are presented in Figure 13 clearly. The overall planning is mainly based on the tactile sensing and position perception system, analyzing and summarizing the mechanism, as well as characteristics of the human hand grasping and mapping to the manipulator grasping.

## 4. Conclusions

Aiming at the grasping, moving, and placing operations of agricultural manipulators, this paper proposes a pre-grabbing planning method based on the understanding of the manual grasping mechanism. Firstly, we designed and fabricated a haptic data acquisition glove incorporating multiple sensors to perform manual grasping simulation. According to the geometric classification of various fruits and vegetables, a grasping gesture library was established and the allocation of virtual fingers in each mode was collated. Then, the mechanism and characteristics during manual gripping were studied in combination with the bending sensors, pressure sensors, and IMU sensor. It includes finger co-movement characteristics (such as inter-finger curvature correlation and inter-joint pressure correlation), slippage detection, and the external interaction law. The conclusion shows that the index, middle, and ring fingers of the human hand have the highest correlation, while the thumb has the highest independence, and there is a linear relationship between the curvature and the radius of the target object. In stable gripping, the gripping force of the DIP joint, PIP joint, and MCP joint decreased sequentially, and the PIP and DIP joints are the most correlated. Mastering the cooperative movement characteristics of the fingers is helpful for the coordinated control of motors. To investigate the law of slip appearance and gripping force signal during gripping, the force signal was processed by wavelet transform to analyze the detailed information in both time and frequency domains. When slip generates, the wavelet transform result will produce significant high-frequency information, which can be used as an important basis for judging whether slip occurs. The real-time slippage detection facilitates timely control of the force and avoids slippage. The data collected by the IMU sensor can be used to analyze the interaction with the external environment during the capture process. When the object is placed in the target plane, a significant pulse signal is generated in the z-axis direction, and when a collision occurs, a significant pulse is generated in the acceleration located in the collision direction. Real-time interaction perception is also a key factor in ensuring smooth crawling and placement operations.

Finally, the robotic gripping planning is developed based on the laws of bending, pressure, and acceleration during manual gripping. According to human grasping experience and object geometric classification, the optimal gripping posture of the manipulator is obtained, and then the initial value of finger bending is set through the curvature model. Meanwhile, the force sensors were used to judge the contact state of the manipulator and the object. The initial grasping force is judged by the force relationship of the three knuckles, and the wavelet transform algorithm is used to detect the occurrence of sliding during the grasping process. Control the servo based on the results of force feedback and sliding feedback, continue to increase the degree of finger curvature, and increase the grasping force without satisfying the three-knuckle force constraint and generating sliding. Otherwise, move the robotic arm to move and place the target object. Finally, judge the external interaction situation according to the acceleration feedback, replan the route when encountering obstacles, and end the capture when placed at the target position. The proposed method is mainly for the flexible and safe non-destructive grasping of fragile and deformable fruits and vegetables, but it can also be applied to non-agricultural robots such as industrial robots and home robots for grasping. The grasping mechanism and the proposed grasping planning method are also common to the above robots.

In the future, the influence of finger surface shape and material on the safe and non-destructive grasping of the manipulator can be further explored. Additionally, the real-time deformation of fruit and vegetable grasping could be combined to build a multi-mode information feedback control model of fragile and easily deformed fruit and vegetable adaptive non-destructive grasping. Meanwhile, we will consider more fruits and vegetables with other shapes or even irregular shapes, so as to improve the robustness of grasping by agricultural manipulator, and further apply the planning strategy to the control research of agricultural manipulators.

## Figures and Tables

**Table 1:** The summary of grasping posture library based on human hands.

Shape	Size		Gesture	Finger	VF Allocation
Sphere	$L<\frac{\pi r}{3}$		Palm-aligned power grab	5	VF1: T VF2: I + M + R VF3: L
	$\frac{\pi r}{3}<L<\frac{2\pi r}{3}$		Proximal phalanx-aligned power grab	5	VF1: T VF2: I + M + R VF3: L
	$L>\frac{2\pi r}{3}$		Precision grab	2	VF1: T VF2: I
cylinder	$L<\frac{\pi r}{3}$	s>width	Palm-aligned power grab	5	VF1: T VF2: I + M + R VF3: L
		s<width	Palm-aligned power grab	$\left(\frac{5s}{width}\right)+1$	VF2: T VF2: I (M/R/L)
	$\frac{\pi r}{3}<L<\frac{2\pi r}{3}$	s>width	Proximal phalanx-aligned power grab	5	VF1: T VF2: I + M + R VF3: L
		s<width	Proximal phalanx-aligned power grab	$\left(\frac{5s}{width}\right)+1$	VF2: T VF2: I (M/R/L)
	$L>\frac{2\pi r}{3}$	s>width	Precision grab	5	VF1: T VF2: I + M + R VF3: L
		s<width	Precision grab	$\left(\frac{5s}{width}\right)+1$	VF2: T VF2: I (M/R/L)
cone	$L<\frac{2\pi r}{3}$		Palm-aligned power grab	5	VF1: T VF2: I + M + R VF3: L
	$L>\frac{2\pi r}{3}$		Precision grab	$\left(\frac{5s}{width}\right)+1$	VF2: T VF2: I (M/R/L)

**Table 2:** Finger curvature under stable grasping of objects with different diameters.

Diameter (cm)	Thumb (°)	Index Finger (°)	Middle Finger (°)	Ring Finger (°)	Little Finger (°)
5.8	67.0804	84.6916	84.84	90.6295	110.0556
6	63.385	85.3011	89.8706	98.9282	104.8757
6.5	62.5786	83.1954	85.3294	87.2685	68.1654
6.8	62.4524	81.5481	81.1659	84.06	66.1351
7.0	50.1655	75.2654	78.6145	45.2645	60.1874
7.4	38.8601	71.8594	74.6515	70.3559	50.0897
7.5	39.1324	71.8624	70.1338	70.6458	54.2648
7.7	39.7285	71.8795	67.8881	70.2586	58.893
8.4	34.1429	55.5814	53.9732	46.116	32.4522
9.1	36.5123	65.8141	65.0091	69.5407	45.0819
9.5	14.7934	75.9312	70.5472	73.6385	41.8194

**Table 3:** Pressure correlation coefficient of the three-finger joint.

Finger	MCP-PIP	MCP-DIP	PIP-DIP
Thumb	0.756522(IP)	\	\
Index finger	0.399683	0.369631	0.593295
Middle finger	0.531877	0.192749	0.651953
Ring finger	0.271122	0.432492	0.837198
Little finger	0.795578	0.607841	0.670623
